# Cytotoxic Effect on Human Myeloma Cells and Leukemic Cells by the* Agaricus blazei* Murill Based Mushroom Extract, Andosan™

**DOI:** 10.1155/2017/2059825

**Published:** 2017-11-07

**Authors:** Jon-Magnus Tangen, Toril Holien, Mohammad Reza Mirlashari, Kristine Misund, Geir Hetland

**Affiliations:** ^1^Department of Acute Medicine, Oslo University Hospital, Ullevål, Kirkeveien 166, 0407 Oslo, Norway; ^2^Institute of Clinical Medicine, University of Oslo, P.O. Box 1171, 0318 Oslo, Norway; ^3^Department of Cancer Research and Molecular Medicine, Norwegian University of Science and Technology (NTNU), St. Olav's University Hospital, Prinsesse Kristinas Gate 1, 7006 Trondheim, Norway; ^4^Department of Immunology and Transfusion Medicine, Oslo University Hospital, Ullevål, Kirkeveien 166, 0407 Oslo, Norway

## Abstract

*Agaricus blazei* Murill is an edible mushroom of the Basidiomycetes family, which has been found to contain a number of compounds with antitumor properties, such as proteoglycans and ergosterol. In the present investigation, we show that the commercial mushroom product Andosan, which contains 82.4%* Agaricus blazei* Murill, together with medicinal mushrooms* Hericium erinaceus* (14.7%) and* Grifola frondosa* (2.9%), has a cytotoxic effect on primary myeloma cells, other myeloma cell lines, and leukemia cell lines* in vitro.* Although the exact content and hence the mechanisms of action of the Andosan extract are unknown, we have found in this investigation indications of cell cycle arrest when myeloma cell lines are cultivated with Andosan. This may be one of the possible explanations for the cytotoxic effects of Andosan.

## 1. Background

*Agaricus blazei* Murill (AbM) is an edible mushroom of the Basidiomycetes family, which grows naturally in the Piedade, coastal rainforest, area in Brazil. Besides being a popular food ingredient, AbM is also used by the local population as a remedy against several diseases, in particular against infection and cancer [1]. After commercial cultivation was started in 1965, AbM has been the subject of extensive scientific investigations, which have revealed strong immunomodulating and antitumor effects [2]. A major part of this research has been conducted on extracts from the mushroom's fruiting body. This part of the mushroom is rich in polysaccharides, in particular *β*-glucans, which have been shown to have strong immunomodulating properties, acting mainly through the stimulation of the innate immune system [3]. The mycelium of AbM has been less well investigated and few details are known concerning its biochemical composition. In the present investigation, a commercial mushroom extract, Andosan, containing 82.4% of* Agaricus blazei Murill,* extracted from the mycelium of the mushroom, has been used. This product also contains two other Basidiomycetes mushrooms,* Hericium erinaceus* (14.7%) and* Grifola frondosa* (2.9%). Antitumor properties have also been attributed to the two latter mushrooms [4, 5]. A recent independent investigation has shown that Andosan, in contrast to extracts from the fruiting body of AbM, contains only a very low amount of polysaccharides (2% of carbohydrates in dry weight, corresponding to 0.009%  *β*-glucan per mL) [6]. On this background, it seems doubtful that the biological effects, which have been observed with Andosan (see below), may be attributed exclusively to the effect of *β*-glucans or other polysaccharides. An immunomodulating effect of Andosan has been reported in several investigations [7, 8]. A proinflammatory effect has been found* in vitro* in human monocytes, human vein endothelial cells [9], and monocyte derived dendritic cells [10]. However, a predominant anti-inflammatory effect was found* in vivo* in healthy volunteers who ingested Andosan for 12 days [11]. In addition, it has been shown that this product ameliorates the skewed Th1/Th2 balance by increasing the Th1 response [7], which is known to have anti-infection and antitumor activities [12]. This effect has been shown to be mediated by small molecules (<12.5 kD) [13], which may easily be taken up from the gut into the blood circulation. Several reports have been published regarding antitumor effects of AbM, the majority using extracts from the fruiting body. It has been shown that *β*-glucans from the fruiting body of AbM have strong tumoricidal effects in preclinical models. The mechanisms involved include enhanced systemic immunity, antioxidant effect, and direct cytotoxic effect by induction of apoptosis [14, 15]. A number of compounds extracted from the fruiting body of the mushroom have been found to be involved in the cytotoxic effects, such as ergosterol [16], the ergosterol derivative agarol [17], agaritine [18], proteoglycans, and other polysaccharides [19, 20]. Reports concerning antitumor effects of extracts from the mycelium of AbM are scarce. A polysaccharide complex from the mycelium of AbM has been shown to have activity against Ehrlich ascites tumor and sarcoma in a mouse model [21]. Furthermore, it has been reported that an ethanol-soluble fraction of Andosan inhibits the activity of the tumor-associated protease, legumain, which may indirectly indicate an antitumor effect [6]. Also, a tumoricidal effect of Andosan has been found by our group in a mouse cancer colon model, as well as a cytotoxic effect correlating with apoptosis, in a human cancer colon cell line [22]. In humans, the use of an AbM extract from the fruiting body as an adjuvant to conventional chemotherapy was found to improve quality of life and increase NK cell activity in patients with gynecological cancer [23].

Multiple myeloma is a malignant disease caused by transformation and clonal expansion of bone marrow plasma cells. The main clinical features are lytic lesions caused by local growth of malignant plasma cells in bones and renal insufficiency due to deposition of paraproteins produced by myeloma cells [24]. Most patients respond well to initial therapy, but relapse occurs in virtually all cases [25]. Our group has previously reported immunomodulating effects of Andosan used in addition to high-dose chemotherapy in patients with multiple myeloma. Furthermore, in the same report, we also documented a direct cytotoxic effect of Andosan on a mouse myeloma cell line [26].

Leukemias constitute the largest group of hematologic malignancies. Despite improvements in therapy, leukemia remains a deadly disease for many patients, especially in the older age group [27]. Research for new treatment principles in order to improve the therapy for multiple myeloma and leukemia is therefore needed.

On this background, we decided to investigate the possible cytotoxic effects of Andosan* in vitro* on primary myeloma cells and human myeloma and leukemic cell lines.

## 2. Materials and Methods

### 2.1. Andosan™

The mushroom extract Andosan was provided by the company Immunopharma AS (organization number 994924273), Oslo, Norway. This commercial product contains extracts from the mushrooms* Agaricus blazei* Murill (mycelium) (82.4%),* Hericium erinaceus* (14.7%), and* Grifola frondosa* (2.9%) and is produced by the company ACE Co. Ltd., Gifu-ken, Japan. The production process comprises fermentation and heat sterilization (commercial information). The lipopolysaccharide (LPS) content was found to be <0.5 pg/mL using the Limulus amebocyte lysate test (COA-MATIC Chromo LAL; Chromogenix, Falmouth, MA, USA). The mushroom extract was stored at 4°C in sterile conditions in dark bottles until use.

### 2.2. Myeloma Cell Lines: Proliferation Assay

The human myeloma cell lines RPMI-8226 and U226 were obtained from the American Tissue Culture Collection (ATCC) (Rockville, MD, USA). INA-6 cells were a kind gift from Dr. Renate Burger, University Medical Center Schleswig-Holstein, Kiel, Germany. The cells were passaged twice a week using media containing 20%–10% fetal calf serum in RPMI-1640 (Sigma-Aldrich, Schnelldorf, Germany) containing L-glutamine (100 *μ*g/mL) and gentamicin (20 *μ*g/mL). For INA-6 cells, recombinant human interleukin-6 (Biosource, Camarillo, CA, USA) 1 ng/mL was added to the media. The cells were grown at 37°C in a humidified atmosphere containing 5% CO_2_. The CellTiter-Glo assay (Promega, Madison, WI, USA), which measures the cells' ATP content, was used to estimate the relative rates of cell proliferation according to the manufacturer's instructions. In short, 10,000 cells were seeded in a total of 100 *μ*L in white opaque 96-well plates and cultured with Andosan at the concentrations 0.5%, 1%, 2%, and 4% or control (PBS) for 72 h. The assay reagent was added to the wells and the plates were mixed for 2 min using a microplate shaker. Then, the plates were left at room temperature for 10 min before luminescence was detected using a Victor 1420 multilabel counter (PerkinElmer Inc., Waltham, MA, USA). The measures were performed in duplicate and the results were noted as levels of ATP synthesis and converted to per cent of controls.

### 2.3. Cell Cycle Analysis

For cell cycle analysis by flow cytometry, 1 × 10^6^ cells (controls and treated myeloma cells) were washed with PBS and fixed by slow addition of 2 mL 100% ice-cold methanol on a mixer and stored at −20°C until analysis. Cell cycle analysis was performed according to Vindeløv et al. [28] (modified) and analyzed on a FACSAria cell sorter (Becton Dickenson, San Jose, CA). Methanol fixed cells were centrifuged (500 g/5 min at 4°C) and the pellet was washed with 1 mL PBS (500 g/5 min at 4°C). The washed cell pellet was resuspended in 200 *μ*L of ice-cold solution A, 200 *μ*L of solution B, and finally 200 *μ*L of solution C. For each solution added, the cells were gently vortexed and incubated for 10 min in the dark. All solutions contained a base of 1 mg/mL trisodium citrate, 1 *μ*l/mL Nonidet P-40, 522 *μ*g/mL spermine, 51 *μ*g/ml Trizma HCl, and 9.5 *μ*g/ml Trizma base. In addition, solution A contained 30.0 *μ*g/mL trypsin, solution B contained 0.1 mg/mL RNase and 0.5 mg/mL trypsin inhibitor, and solution C contained 0.28 mg/mL propidium iodide (PI) and 1.16 mg/mL spermine. Cell nuclei were kept on ice in the dark until being analyzed by flow cytometry. 10,000 nuclei were recorded. Aggregated nuclei were excluded in a dot plot displaying a pulse width of PI (PI-w linear scale) and pulse height of PI (PI-h linear scale). Single nuclei were displayed in a histogram of pulse area of PI (filter 616/23). Flow cytometric analysis was performed with FACSDiva software version 6.0 (Becton Dickenson, San Jose, CA). The cell lines and myeloma cell lines INA-6, RPMI-8266, and U226 were cultured with the addition of Andosan 10% or with PBS (controls). The results were noted as per cent of cells in cell cycle phases sub-G1, G1, S, and G2, respectively. For each cell line, flow cytometric analysis was performed five times and the mean values were noted.

### 2.4. Leukemia Cell Lines: Cell Proliferation Assay

The human leukemic cell lines KG1a, HL 60, and Meg 01 were obtained from Deutsche Sammlung von Mikroorganismen und Zellkulturen GmbH (German Collection of Microorganisms and Cell Cultures), Braunschweig, Germany. The cells were cultured in RPMI-1640 medium (ATCC 30-2001) supplemented with 10% fetal bovine serum (ATCC, cat. number 3020) and 1% antibiotic mix (Sigma A5955) and maintained in a humidified atmosphere with 5% CO_2_ at 37°C. The media were changed twice a week. For the cytotoxic assay, the cells were seeded in 24-well plates at a density of 7.5 × 10^4^ cells/mL and treated with various concentrations of Andosan (5.0% and 10.0%), or a matched concentration of PBS as a control, for 96 hrs. The total number and percent viable cells were counted by NucleoCounter using the NucleoCassette kit (ChemoMetec, Allerød, Denmark) according to the manufacturer's manual. For controls and each concentration of Andosan, the mean of five parallel measurements was noted. The results were converted to per cent of the number of viable cells in controls (100%).

### 2.5. Primary Myeloma Cells: Cell Proliferation Assay

Primary CD138+ myeloma cells from ten patients were isolated from bone marrow specimens included in the Norwegian Myeloma Biobank using RoboSep automated cell separator and human CD138 Positive Selection Kit (Stem Cell Technologies, Grenoble, France). The study was approved by the Regional Ethics Committee (approval 2016/828/REK Midt) and all patients gave informed consent. To determine the effect of Andosan on the viability of primary myeloma cells, 5000 cells were seeded per well in 96-well plates. The cells were cultivated in 2% heat-inactivated human serum (HS; Blood Bank, St. Olav's University Hospital, Trondheim, Norway) in RPMI and 2 ng/mL interleukin-6 (IL-6) (Gibco, Thermo Fisher, Waltham, MA, USA). Andosan was added to a final volume of 200 *μ*L per well at the concentrations 0.5%, 1%, 2%, and 4%. All samples were run in duplicate. The effect on cell viability was determined by counting the number of viable myeloma cells in each well after 72 h incubation using ScanR automated image acquisition and analysis (Olympus, Hamburg, Germany) as previously described [29]. The measures were performed in duplicate and the results were converted to per cent of the number of viable cells in controls (100%).

### 2.6. Peripheral Blood Mononuclear Cells

Peripheral blood mononuclear cells from three healthy blood donors were grown in the RPMI-1640 medium supplemented with 10% heat-inactivated fetal bovine serum (FBS) (ATCC, cat. number 30-2020) and 1% Antibiotic Antimycotic Solution (Sigma, A5955), and maintained in a humidified atmosphere with 5% CO_2_ at 37°C. The media were changed twice a week. For cytotoxicity assay, the cells were seeded in 24-well plates at a density of 7.5 × 10^4^ cells/mL with various concentrations of Andosan (0.5%, 1%, 5%, and 10%) or PBS (control) for 72 hrs. The total number and percent viable cells were counted by NucleoCounter using the NucleoCassette kit (ChemoMetec, Allerød, Denmark), according to the manufacturer's manual.

*Statistics*. The differences between the means of the percent of viable cells in controls (100%) and the means of the percent of viable cells in cultures with Andosan 4% (myeloma cell lines and primary myeloma cells) or Andosan 10% (leukemia cell lines and peripheral blood mononuclear cells) were calculated by the paired samples *t*-test using the IBM SPSS 23 statistical computer program. *p* values below 0.05 were considered statistically significant. In primary myeloma cells and myeloma cell lines, the correlations between Andosan concentration and viability of the cells were calculated by Pearson's product moment correlation. For cell cycle analysis, a comparison of the percentage of cells in sub-G1 phase and in G1 phase of the cell cycle in cells cultured with Andosan and cells cultured with PBS (controls) was made with the Bonferroni method.

## 3. Results

### 3.1. Primary Myeloma Cells

The results from two patients were excluded from the analysis because of initial low cell viability (20% and 13%, resp.). The results from the remaining eight patients were considered to be evaluable. In seven patients, a dose-related inhibitory effect of Andosan was noted (correlation coefficient: −0.71 to −0.99), with a reduction of viable myeloma cells from 19.5% to 82.4% in cultures with 4% Andosan compared to controls. In contrast, in one patient (number 244), the number of viable cells increased during culture with Andosan, although there was no correlation (correlation coefficient: 0.06) (Table 1). Comparison of the means of controls versus the means of cell cultures with Andosan 4% showed a statistically significant difference (*p* = 0.01).

### 3.2. Myeloma Cell Lines

**Figure 1:**
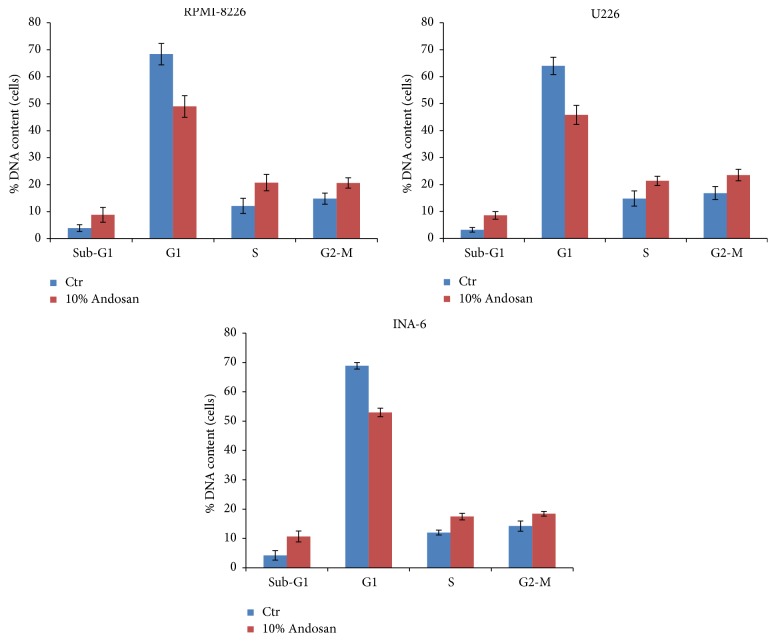
Cell cycle analysis. The figure shows the percentage of cells cultured with either Andosan 10% or PBS (controls) in different cell cycle phases (sub-G1 phase, G1 phase, S-phase, and G2 phase). The percentage of cells was higher in the sub-G1 phase (*p* < 0.002) and lower in the G1 phase (*p* < 0.001) in cells cultured with Andosan 10% compared to controls (paired *t*-test), which is suggestive of cell cycle arrest in these cells.

In the myeloma cell lines RPMI-8226, U226, and INA-6, a dose-related inhibitory effect of Andosan was found (correlation coefficient: −0.94), expressed as per cent reduction of ATP content compared to controls. Comparison of the means of the controls versus the means of cells cultured with Andosan 4% showed a statistically significant difference (*p* = 0.02) (Table 2). Furthermore, in a cell cycle study, the percentage of cells was higher in sub-G1 phase (*p* < 0.002) and lower in the G1 phase (*p* < 0.001) in cells cultured with Andosan 10% (paired *t*-test) compared to controls, which is suggestive of cell cycle arrest in these cells (Figure 1).

### 3.3. Leukemia Cell Lines

In leukemia cell lines, a comparison of the means of the controls versus the means of the cells cultured with Andosan 10% showed a significant statistical difference (*p* = 0.02) (Table 3).

### 3.4. Peripheral Blood Mononuclear Cells

A comparison of the means of the controls and the means of the cells cultured with Andosan 10% showed no statistical difference (Table 4).

## 4. Discussion

This study shows a predominantly dose-related cytotoxic effect of Andosan on primary myeloma cells and human myeloma and leukemic cell lines* in vitro*. These results are in line with previous reports of cytotoxic effects of different compounds (*β*- glucans, proteoglycans, ergosterol, and agaritine) extracted from AbM preparations from the fruiting body, on different malignant tumors, both* in vitro* and in animal models [14–20]. In particular, it has been shown that an extract of the fruiting body of* Agaricus blazei* Murill had an antitumor effect in a mouse myeloma model, when given together with a marine phospholipid [30]. In the case of Andosan, which is a commercial mushroom extract where the exact content is not known, a firm conclusion regarding the mechanism behind the cytotoxic effects is not possible. However, it is remarkable that indications of cell cycle arrest were found when the myeloma cell lines RPMI-8226, U226, and INA-6 were cultivated with Andosan. We and others [19, 20, 22] have previously found that AbM extracts can have cytotoxic effects on tumor cells by induction of apoptosis. Also, it has been documented that an ethanol-soluble fraction of Andosan inhibits the tumor-associated protease legumain in the murine macrophage-like cell line RAW 264.7 [6]. This may indirectly indicate an antitumor effect of this fraction, as legumain is secreted by a number of malignant cells [31]. In fact, in the mouse model for colon cancer, Andosan did also induce reduced expression of legumain in the intestinal wall [6]. Moreover, it increased levels of Th1 cytokine IL-12 in addition to proinflammatory cytokines [22]. The latter is in contrast to what we have usually observed in humans consuming Andosan. However, it agrees with our previous* in vitro* finding of Andosan-induced NF_K_-B activation via stimulation of TLR2 in dendritic cells [32]. Furthermore, the possibility of a synergistic effect between the three mushrooms contained in Andosan—*Agaricus blazei* Murill,* Grifola frondosa*, and* Hericium erinaceus*—may also be taken into consideration. Importantly, Andosan did not have a toxic effect neither on human peripheral mononuclear cells (PMNC) nor on normal human hematopoietic stem cells (G. Hetland et al., unpublished results).

The cytotoxic effects of Andosan found in this investigation on primary myeloma cells and myeloma cell lines are particularly interesting in light of the previously reported immunomodulating effects mostly associated with antitumor properties, when this product was used as an adjuvant treatment in myeloma patients undergoing high-dose chemotherapy [26]. It therefore seems plausible that the mushroom extract may have both cytotoxic and immunomodulating antitumor mechanisms of action in myeloma. Further investigations are needed in order to clarify whether Andosan may have a role in the treatment of multiple myeloma.

## Figures and Tables

**Table 1:** Cytotoxic effect of Andosan on myeloma cells from 8 patients. The numbers of viable cells after 72 hrs of culture were noted and converted to per cent of controls (100%). Mean: mean of duplicates; SE: standard error. Comparison of means of controls versus means of cultures with Andosan 4% showed a statistically significant difference (*p* = 0.01).

Patient number	Control	Andosan 0.5%	Andosan 1.0%	Andosan 2.0%	Andosan 4.0%
139					
Mean	100	93.69	99.12	97.61	80.47
SE	4.04	1.35	1.11	2.11	0.44
244					
Mean	100	117.91	122.5	114.15	110.22
SE	1.33	2.07	2.1	4.19	2.13
969					
Mean	100	65.73	62.3	55.42	52.37
SE	1.95	2.18	2.22	2.66	2.39
185					
Mean	100	86.6	83.14	73.71	52.28
SE	0.88	1.38	0.66	1.82	0.77
409					
Mean	100	69.24	55.75	50.6	17.66
SE	1.5	2.05	2.05	2.05	2.18
715					
Mean	100	90.35	86.8	84.12	73.11
SE	1.54	1.73	2.72	0.92	1.21
2925					
Mean	100	91.81	90.92	80.78	54.27
SE	0.87	3.4	2.19	1.88	1.88
355					
Mean	100	82.22	85.21	78.34	70.77
SE	2.17	2.64	2.39	3.15	2.95

**Table 2:** Cytotoxic effect of Andosan on myeloma cell lines. The effects are measured as levels of DNA synthesis and converted to per cent of controls (100%). Mean: mean of duplicate experiments. Comparison of the means of controls compared to means of cell lines cultured with Andosan showed a statistically significant difference (*p* = 0.02). SE: standard error.

Cell type	Control	Andosan 0.5%	Andosan 1%	Andosan 2%	Andosan 4%
INA-6					
Mean	100	93.9	71.67	66.06	49.46
SE	0.24	1.89	2.15	1.27	1.31
RPMI-8221					
Mean	100	94.88	88.53	77.21	66.12
SE	1.92	1.95	1.07	2.07	2.22
U226					
Mean	100	96.92	87.13	79.7	69.27
SE	0.93	1.24	1.95	2.07	2.22

**Table 3:** Cytotoxic effect of Andosan on leukemia cell lines. Mean: mean of five experiments; SE: standard error. Comparison of the means of control with means of cells cultured with Andosan 10% showed a statistically significant difference (*p* = 0.02).

	Controls	Andosan 5%	Andosan 10%
HL 60			
Mean	100	83.83	76.16
SE	0	2.6	3.13
Kg1a			
Mean	100	84.81	74.6
SE	0	1.92	2.3
Meg			
Mean	100	92	83
SE	0	3.16	4.1

**Table 4:** Effect of Andosan on peripheral blood mononuclear cells. Mean: mean of 5 experiments; SE: standard error. Comparison of the means of controls versus means of Andosan 10% showed no statistical difference (*p* = ns).

	Controls	Andosan 0.5%	Andosan 1%	Andosan 5%	Andosan 10%
Mean	100	97.70	97.02	97.00	95.22
SE	0.97	0.96	1.07	0.62	1.39

## Data Access

The datasets used and analyzed during the current study are available from the corresponding author on reasonable request.
